# Notes and Observations in General Practice

**Published:** 1868-08-15

**Authors:** John Forman

**Affiliations:** Edinburgh


### NOTES AND OBSERVATIONS IN GENERAL PRACTICE.

BY JOHN FORMAN, M.D., EDINBURGH.

### Paper III.—Uterine Diseases.

Case 1. *Menorrhagia.* Mrs. G——; aged 23 years; short stature, pale and thin; mother of three children—youngest fifteen months old; catamenia reappeared five months ago. The case came under my care April 5th, 1868, when she stated that she had been under treatment for profuse menorrhagia for upwards of three months without benefit—her excessive catamenia continuing from ten to fourteen days every month, which had reduced her general strength very considerably, and made her nervous and irritable. She slept badly, had sick headache, and impaired appetite.

A speculum examination revealed both lips of the *os uteri* hypertrophied, gaping, and surrounded with prominent granulations, extending into the cervical canal, which bleed freely when touched. The sound did not detect any polyphoid or other tumor in the uterus, which was exquisitely tender, felt relaxed, and bled freely from the internal wall. The uterus was injected with a weak solution of perchlorlide of iron to check the hæmorrhage. On the following day, a solution of nitrate of silver, two grains to the ounce of water, was injected, the granular portion of the os and cervix uteri painted over with acid, nitrate of mercury, and painted over once a week; the nitrate of silver injection every four days; the vagina daily injected with a solution of alum. She took acetate of lead and opium pills, and a tonic mixture, containing quinine and chloride of iron.

Her menorrhagia ceased, the catemania becoming normal five weeks after the first injection of nitrate of silver, which was repeated four times. The morbid condition of the *os* and *cervix uteri* disappeared shortly afterwards. The acid, nitrate of mercury was applied thereto, when solid nitrate of silver was lightly applied twice a week until cicatrization was completed. The alum solution used daily to cleanse the vagina, and avoid the accumulation of muco-purulent matter. A piece of lint with a tape attached soaked in the same liquid, and introduced into the vagina, to prevent undue irritation of the walls.

REMARKS.—This is an example of a numerous class of patients, which are ever and anon coming under the notice of the practitioner of medicine, and I may add that menorrhagia, with or without ulceration of the *os uteri*, combined more or less with leucorrhœa, is one of the most distressing uterine ailments, which assail the gentler sex. Apart from the more serious causes of the ailment, such as cancerous, fibrous and polypoid tumours, we very frequently meet with it arising from a morbid condition of the uterus and ovaries, which, when properly recognized, are very successfully corrected. The most frequent lesion causing the uterine hæmorrhage, we find it to be an eroded condition of the mucous membrane of the uterus. This may exist, as we have already remarked, with or without a granular state of the *os uteri*.

The cause of this distressing form of menorrhagia may no doubt be sought for in some peculiar state of the general system unfavorable to the proper development of the periodic decidual membrane, and as a natural consequence the mucous membrane of the uterus assumes a disposition very similar to granular inflammation in other parts of the mucoid system. If this view be correct, our general treatment of such cases should be vigorously supported with direct local remedies. What can be more hopeless and discouraging, than to depend solely upon general measures in cases of *os uteri* ulceration, daily experience proves. The same argument is equally strong in the case of erosion in the ulceroid cavity, yet how many patients do we come across, who have been drenched with medicine for months and years, who may ask in despair, Must this miserable state haunt them their life-long existence? Happily, experience urges us to answer in the negative. Where patient local perseverance in judicious local treatment is adopted, in conjunction with general restorative remedies, the physician's effort's will rarely disappoint him in this class of ailments. He will almost invariably be rewarded with the high satisfaction of being the instrument of extinguishing a vast amount of female suffering and misery.

### Paper IV.—Os Uteri Ulceration.

Case.—History. Miss G——; aged 30 years; seamstress; tall, spare frame; dark complexion.

March 18, 1868. Patient states that she has suffered pain from leucorrhœa discharge; with accompanying discomforts of heat, scalding, and external excoriation for two years, and has been under treatment nearly all that time, without any benefit

—rather the reverse. She had some liquid painted over the womb, which did not cause her any uneasiness. She has also taken a quantity of medicine. She complains of general weakness, languor, and impaired appetite; feels very nervous, and is easily excited; her catamenial periods are regular, with an augmented overflow, continuing from five to seven days.

Speculum examination reveals considerable hypertrophy and fungoid; ulceration of both lips of the *os uteri; cervix* slightly dilated, and, like the *os*, of a deep, red, angry appearance, bathed in a muco-purulent sensation, streaked with blood. The walls of the vagina are considerably inflamed and tender. The sound does not indicate any abnormality in the uterine cavity.

*Treatment.*—The uterus injected with two grains of *Nitrate of Silver*, dissolved in half an ounce each of glycerine and water. The *os* and *cervix uteri* freely cauterized with solid *Chloride of Zinc.* The vagina to be injected twice a day with the *Nitrate of Silver* solution already mentioned; also a piece of lint with a piece of tape attached, soaked in the same liquid, and introduced into the vagina after each injection. To take a course of iron with *Bromide of Potassium* in a bitter infusion, and a nutritious diet.

REMARKS.—Four applications of the *Chloride of Zinc*, produce healthy granulations of the *os uteri*, which cicatrize eight weeks after the first application. The *Nitrate of Silver* injection and lint plug were used daily during that period, when the vaginal walls assumed their natural aspect, and her general health simultaneously improved—the discontinuance of the irritating discharge from the vagina very materially increasing her general comfort. As a more bracing tonic, *Strychnia* was now substituted for the *Bromide of Potassium*, in conjunction with *Quinine* and *Iron.*

In the treatment of *os uteri* disease, *Caustic Potass.* was for a long time quite a favorite application with some of our leading obstetric practitioners, who frequently used it extravagantly in hypertrophied *os uteri*. Experience has shown, however carefully this caustic is applied and counteracted, when the desired cauterization has been effected, its rapidly deliquescent nature renders it exceedingly liable to spread over a portion of the vaginal walls, frequently terminating in adhesion of the *os uteri* to one side or the other, and proving a tedious obstacle in subsequent pregnancies. *Chloride of Zinc* is a much more manageable agent, when prepared in suitable sized sticks. It is much less deliquescent, and equally effective in fungoid ulceration, and the reduction of hypertrophied *os uteri*. At the same time it possesses the advantages of producing a sore which heals more kindly.

In simple ulceration, the *Acid*, *Nitrate of Mercury*, or *Carbolic Acid*, are to be preferred. When properly applied, they do not cauterize deeply, and heal quickly. Lint, soaked in a weak solution of *Nitrate of Silver* or *Carbolic Acid*, and introduced into the vagina after it has been injected with a mild astringent solution, materially adds to the patient's comfort by keeping the irritated sides apart, and at the same time facilitates the reproduction of healthy epithelium.

### Paper V.—Leucorrhœa.

Case.—History. Mrs. M——; aged 25 years; spare frame and fair complexion. March 26th, 1868. States that she has been married over two years; has not been pregnant. Prior to her marriage she enjoyed excellent health; had regular catamenias without leucorrhœa. About six months after her marriage, a white discharge appeared and gradually increased in quantity, by and by becoming continuous, causing her great discomfort, scalding, excoriation, etc. When her general health became affected she applied to a physician; had medicines and a vaginal injector prescribed, which she used for some time without benefit.

Speculum shows the *os uteri* to be of normal size and free of abrasion; *cervix* plugged with an albuminous secretion, vagina and labia of a deep red color, excoriated and extremely sensitive; uterine sound, with considerable difficulty; uterus of normal size, but very sensitive; sound coated with albumenial secretion.

TREATMENT.—The day after the cessation of her catamenial period, a small sponge tent was introduced into the *cervix uteri* to dilate it sufficiently to admit a syringe freely, when the uterus and vagina were freely injected with tepid water, and followed with an injection of two grains of *Nitrate of Silver*, dissolved in half an ounce each of glycerine and water. This induced smart uterine pains for about two hours, when it ceased. The vagina was injected daily with tepid water, and a pessary introduced containing two grains of *Oxyde of Zinc*. She was ordered *Quinine* and *Iron* with *Phosphoric Acid* in a bitter infusion. The uterine injection was not repeated, the pessaries were continued for two weeks, when the leucorrhœa discharge was much less. A weak alum solution was now injected daily and continued until the 2nd of May, when she reported herself quite well.

REMARKS.—Independent of uterine tumors, and carcinoma or ulceration of the *os* and *cervix uteri*, we frequently meet with leucorrhœa of a troublesome and distressing nature. This morbid secretion may arise from chronic congestion of the ovaries, or the lining coats of the uterus, or an inflammatory condition of the mucous membrane of the vagina or labia; and in advanced or neglected cases we find congestion giving place to granulations. Causes like these, singly or combined, are of very frequent occurrence. I have already remarked that the uterine cavity was only once injected with *Nitrate of Silver* in this coat, in the belief that the mucous coat was only congested. Where this has advanced to excessive exfoliation and granulation, we invariably have augmented catamenial flow with increased general distress. In such cases it is found necessary to repeat the injections, and, in some instances, to augment their strength, to reduce the granular points, and induce the formation of healthy membrane. From whatever lesion leucorrhœa springs, its source must be recognized through the medium of the speculum, or sound, which are indispensable guides in our search for the seat of mischief, to enable us to form a correct diagnosis upon which to assure the basis of disease, by clearing away the *debris* before we commence to rear the superstructure of rational treatment. Ignoring or neglecting this, and depending upon general measures alone, is the most forlorn task the obstetric practitioner can encounter.

### Paper VI.—Case of Albuminuria, treated with Nitrite of Urea.

History.—Dr. W—— desired me to visit Mrs. ——, in consultation with him on the 2nd of June ult., whose history was as follows:

Mrs. H——; aged 29; carpenter's wife; tall, nervous and cachectic; mother of four children, the youngest 18 months old. She states that fifteen weeks ago she was seized with severe pain in the lower part of her back, and became very ill, when she called a physician, who told her she was suffering from an attack of intermitting fever; and subsequently, that the severe persisting pain in the back was the result of rheumatism. Her feet soon began to swell, which extended to her legs, thighs and body. Her breathing became embarrassed, compelling her to be supported upright in bed. She became so weak that she required to be lifted from side to side in bed. About four weeks after the onset of her illness, an abscess broke over the pained part in the lower region of her back, and discharged a large quantity of matter. It has continued alternately to heal, re-form and discharge, ever since. The swelling in her chest has diminished of late, but her thighs, legs and feet continue the same.

A short time ago her medical attendant brought another physician to see her in consultation. Immediately after this, her husband solicited Dr. W—— to attend her.

SYMPTOMS.—Pulse weak, 85; integument over throat œdematous; lungs normal; heart's action slightly accelerated, otherwise normal; abdomen distended; umbilical region dull on percussion; iliac tympanitis, thighs, legs and feet œdematous, pitting deeply on pressure; circumference of left leg fifteen and a half inches—right one sixteen inches. She sleeps a great deal; does not have headache; tongue clean; appetite impaired; bowels regular; urine scanty, pale and turbid, of strong acid reaction; sp. gr. 10.05, and forms an albuminous mass, with heat and nitric acid; shows an abundance of tube casts; her catamenia has not appeared since her illness began; no pregnant signs. There is an abscess discharging over lower lumbar spine; no carious vertibræ.

TREATMENT.—A piece of India rubber drainage tube was introduced into the immediate vicinity of the abscess, when a large quantity of serum, tinged with blood, flowed. She was ordered wine and a suitable nourishing diet, with the following medicine:

| | | |
|---|---|---|
| ℞. | *Urea Nitrite* . | ℈iv. |
| | *Potass. Acetat.* . | ʒij. |
| | " *Bicarb.* . | ʒiv. |
| | *Tinct. Digitalis* . | ʒiv. |
| | *Decoct. Uvæ. Ursi* . | ℥xx. |

M. Capt. coch. magna. et ter. in die.

℞. *Elixir. Valer. Ammon. et Quina* ʒij. *bis die sumenda.*

July 4th. Patient's general condition considerably improved; urine more abundant, less cloudy, only slightly acid; sp. gr. as before; increased cloudiness, with heat, moderate coagulation, and nitric acid.

July 9th. Patient able to move without assistance, and sit up with comfort; appetite improved, sleeps well, and expresses herself as much better; pulse stronger and less accelerated; urine clear, neutral, and quite free from albumen; œdema in the extremities much less.

Discontinue Mixture to have *Potass. Bitart.* ʒj. three times a day, with bitter tonics and nourishing regimen.

REMARKS.—This a good example of a case of albuminuria resulting from acute nephritis. The most remarkable feature was the entire disappearance of albumen from the urine. In the course of three weeks after treatment with *Nitrite of Urea* was commenced, five grain doses induced slight nausea. After ten days the dose was reduced to three grains, and the *Potass.* withdrawn. When the urine became normal, *Bitartrate of Potass.* was prescribed to redeem the remaining œdema of the lower extremities.

When *Bitartrate of Potass.* can be procured pure, it is certainly one of the best diuretics we possess. In full doses is apt to excite, in some irritable mucoid diathesis, undue action of the alimentary canal. This, in turn, may be regulated with the addition of gallic acid, or some other astringent. The case continues under treatment. Should any novel feature arise, it may be referred to again.

